# Health Benefits of Key Constituents in *Cichorium intybus* L.

**DOI:** 10.3390/nu15061322

**Published:** 2023-03-08

**Authors:** Mihail Lucian Birsa, Laura G. Sarbu

**Affiliations:** Department of Chemistry, Alexandru Ioan Cuza University of Iasi, 11 Carol I Blvd., 700506 Iasi, Romania

**Keywords:** *Cichorium intybus* L., antioxidant, inulin, cichoric acid, cholorogenic acid

## Abstract

The genus *Cichorium* (*Asteraceae*) that originates from the Mediterranean area consists of six species (*Cichorium intybus*, *Cichorium frisee*, *Cichorium endivia*, *Cichorium grouse*, *Cichorium chico* and *Cichorium pumilum*). *Cichorium intybus* L., commonly known as chicory, has a rich history of being known as a medicinal plant and coffee substitute. A variety of key constituents in chicory play important roles as antioxidant agents. The herb is also used as a forage plant for animals. This review highlights the bioactive composition of *C. intybus* L. and summarizes the antioxidant activity associated with the presence of inulin, caffeic acid derivatives, ferrulic acid, caftaric acid, chicoric acid, chlorogenic and isochlorogenic acids, dicaffeoyl tartaric acid, sugars, proteins, hydroxycoumarins, flavonoids and sesquiterpene lactones. It also covers the plant’s occurrence, agriculture improvement, natural biosynthesis, geographical distribution and waste valorization.

## 1. Introduction

The genus *Cichorium*, part of the dandelion family *Asteraceae*, consists of six species: *C. intybus*, *C. frisee*, *C. endivia*, *C. grouse*, *C. chico* and *C. pumilum*. The origin of these species is the Mediterranean region, but they also may be cultivated worldwide in temperate and semi-arid areas [1]. *C. intybus* L., commonly known as chicory, is a perennial herbal plant most often bearing bright blue flowers that has been grown since ancient times. Besides the medicinal application of this plant, there are several other uses of *C. intybus* L., including the industrial extraction of inulin, as a coffee substitute, or as animal food. Moreover, the leaves of the plant can be consumed raw or cooked [2,3].

**Figure 1:**
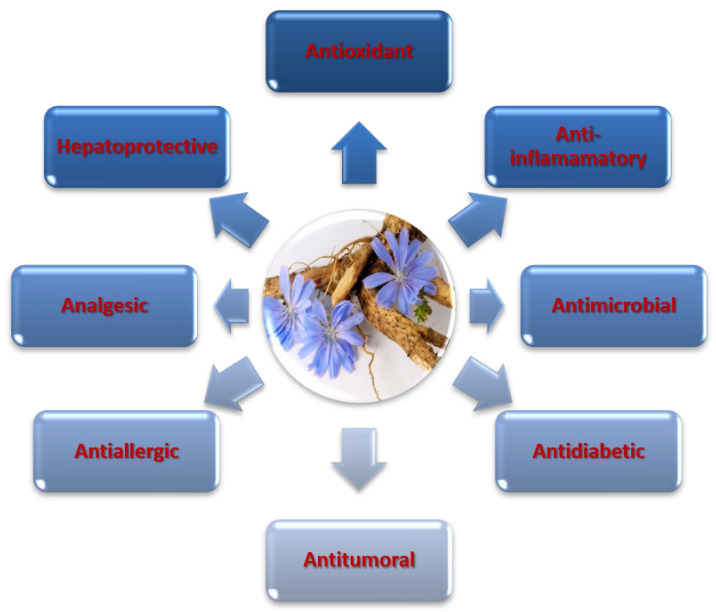
Pharmacological properties of *C. intybus* L.

In addition to the above-mentioned benefits, *C. intybus* L. possesses numerous biological properties, including antioxidant, hepatoprotective, anti-inflammatory, antidiabetic, antimicrobial and tumor-inhibitory activity [4,5]. Inulin and specialized metabolites such as hydroxycinnamic acids, coumarins, flavonoids and sesquiterpene lactones that are located in the different parts of *C. intybus* L. could be responsible for these biological properties [6]. Hydroxycinnamic acid derivatives, especially hydroxycinnamoyl esters, are extensively distributed in the plant kingdom [7]. They are phenolic compounds that are well known for their antioxidant properties and could play a role in the prevention of various diseases associated to oxidative stress. Since sucrose and fructans are known for their radical scavenging properties in plant cells [8], it has been suggested that the in vitro antioxidant activity of chicory may be attributed not only to the phenolic derivatives. Pharmacological properties of *C. intybus* L. are summarized in Figure 1.

Chicory as a functional food has been studied by the food industry in the form of root flour [9]. Functional foods, also known as nutraceuticals, can be widely described as processed foods that provide medical or health benefits as well as a reduction in disease risk. There is evidence that nutraceuticals extracted from plants can significantly reduce the incidence of chronic diseases [10].

Chicory is also a valuable vegetable crop worldwide for making coffee substitutes from roots. In recent decades, coffee substitutes have become a popular alternative to usual coffee brews. This trend is due to the absence of caffeine, a rich bioactive composition and the specific sensory properties of these brews. The combination of roasted chicory roots and barley has provided a product with a high antioxidant capacity [11,12].

## 2. Chemistry

Phytochemical analysis indicated that the different parts of *C. intybus* contained inulin (**1**), caffeic acid derivatives (**2**) such as ferrulic acid (**3**), caftaric acid (**4**), chicoric acid (**5**), chlorogenic acid (3-CQA, **6**), isochlorogenic acid (3,5-diCQA,) (**7**), dicaffeoyl tartaric acid, sugars, proteins, hydroxycoumarins (e.g., **8**, **9**, **12**), flavonoids (e.g., **10**, **11**), terpenoids, sesquiterpene lactones (e.g., Crepidiaside A) (**13**), alkaloids, steroids, oils, volatile compounds, vitamins and polyynes (Table 1).

In nature, inulin (**1**) is the second most abundant storage carbohydrate after starch [13]. Chemically, inulin is an unbranched polysaccharide affiliated to the class of fructans. Inulin consists of around 30 β-fructosyl fructose units (presented in furanose form) usually with glucopyranose unit-reducing ends linked through β-1,2-glycosidic bonds [14,15]. The moderate average degree of polymerization and its availability made this polysaccharide of interest for human health [16]. Thus, inulin has been utilized as either functional food [17] in the cosmetic industry [18] or for biomedical applications [19]. The pharmaceutical and physiological implications of inulin have been reviewed [20]. Important amounts of inulin have been reported for fresh (ca. 68%) and dried (ca. 98%) chicory, as well as for other compounds [21].

A survey on the chemical transformation of inulin and their industrial application has been provided [22]. The synthesis of *N*-(aminoethyl)inulin monosubstituted at *C*-6 has been reported and its potential antioxidant capacity against hydroxyl radicals has been investigated at different concentrations [23]. *N*-(Aminoethyl)inulin proved to be a useful precursor for the chemical transformation of inulin.

Cichoric acid (**5**) is a derivative of both cafeic and tartaric acids. Cichoric acid is a phenolic derivative with important antioxidant properties [24]. Its high antioxidant activity is due to the presence of a catechol ring in the structure of this bioactive compound [25]. An overview on the effects of chicoric acid as a functional food ingredient has been recently presented [26]. It has been found that, in chicory, the major phenolic acid is chicoric acid, with the highest concentration (1692.33 g/g d.w.) [27]. On the other hand chichoric acid complexes with Cu(II), Ni(II), Co(II) and Zn(II) have been reported to exhibit higher antimicrobial activity than the ligand alone, which might be the result for its increased absorption [28].

Using high-performance liquid chromatography, the amounts of caffeic acid and six chlorogenic acids, 3-CQA **6**, 4-CQA, 5-CQA, 3,4-diCQA, 3,5-diCQA **7** and 4,5-diCQA, have been analyzed in several vegetables consumed in Brazil [29]. Chicory and collard greens have been identified as the major source for monocaffeoylquinic acids 3-, 4- and 5-CQA.

Moreover, although not directly related, it should be noted that multiple biological roles have been attributed to caffeoylquinic esters, which were shown to be more resistant to microbes [30]. Caffeoylquinic derivatives have been also demonstrated as key intermediates in the synthesis of lignin [31].

Interestingly, the tissue distribution of tartaric acid esters has been highlighted [32,33]. These have been shown to be mainly concentrated in the aerial part, whereas 3,5-dicaffeoylquinic acid (3,5-diCQA, **7**) was mainly located in the root [34]. Chlorogenic acid (3-CQA, **6**) has been found to be evenly distributed in all organs. The biosynthesis of hydroxycinnamates in *C. intybus* L. has been described, and it was revealed that two types of enzymes are involved in the synthesis and accumulation of CQA.

The main constituents of *C. intybus* L. are presented in Table 1.

**Table 1:** Main chemical constituents of *Cichorium intybus* L.

Compound No.	Common Name/IUPAC Name	Structure	Ref
**1**	Inulin	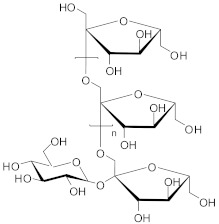	[35]
**2**	Caffeic acid/ (*E*)-3-(3,4-Dihydroxyphenyl)prop-2-enoic acid	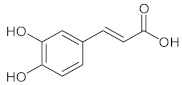	[36]
**3**	Ferulic acid/(2*E*)-3-(4-Hydroxy-3-methoxyphenyl)prop-2-enoic acid	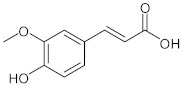	[37]
**4**	Caftaric acid/ (2*R*,3*R*)-2-{[(2*E*)-3-(3,4-Dihydroxyphenyl)prop-2-enoyl]oxy}-3-hydroxybutanedioic acid	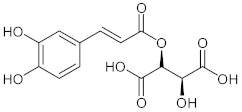	[38]
**5**	Chicoric acid/ (2*R*,3*R*)-2,3-Bis{[(2*E*)-3-(3,4-dihydroxyphenyl) prop-2-enoyl]oxy}butanedioic acid	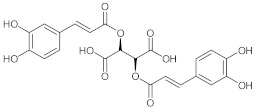	[39]
**6**	5-Caffeoylquinic acid (Chlorogenic acid)/ (1*S*,3*R*,4*R*,5*R*)-3-{[(2*E*)-3-(3,4-Dihydroxyphenyl)prop-2-enoyl]oxy}-1,4,5-trihydroxycyclohexane-1-carboxylic acid	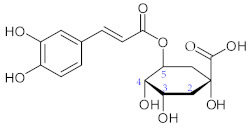	[40]
**7**	3,5-Dicaffeoylquinic acid (Isochlorogenic acid A)/ (3*R*,5*R*)-3,5-Bis{[(2*E*)-3-(3,4-dihydroxyphenyl)prop-2-enoyl]oxy}-1,4-dihydroxycyclohexanecarboxylic acid	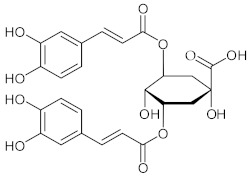	[38,41]
**8**	Aesculetin/ 6,7-Dihydroxy-2*H*-1-benzopyran-2-one	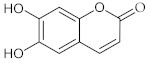	[42]
**9**	Aesculin/ 6-(*β*-D-Glucopyranosyloxy)-7-hydroxy-2*H*-1-benzopyran-2-one	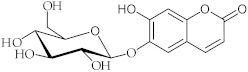	[42]
**10**	Luteolin/ 2-(3,4-Dihydroxyphenyl)-5,7-dihydroxy-4*H*-1-benzopyran-4-one	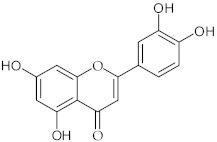	[43]
**11**	Isoquercetin/ 3-(*β*-D-Glucopyranosyloxy)-3′,4′,5,7-tetrahydroxyflavone	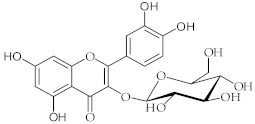	[38]
**12**	Ellagic acid/ 2,3,7,8-Tetrahydroxy (1)benzopyrano[5,4,3-*cde*](1)benzopyran-5,10-dione	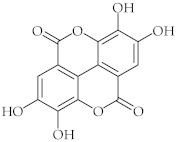	[44]
**13**	Crepidiaside A/ (3*aS*,9*aS*,9*bS*)-6-Methyl-3-methylidene-9-({[(2*R*,3*R*,4*S*,5*S*,6*R*)-3,4,5-trihydroxy-6-(hydroxymethyl)oxan-2-yl]oxy}methyl)-3*aH*,4*H*,5*H*,9*aH*,9*bH*-azuleno[4,5-*b*]furan-2,7-dione	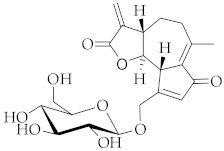	[45]
**14**	Cyanidin	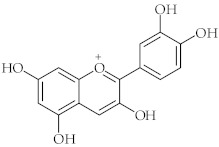	[46,47]

A large number of flavonol derivatives and phenolic acids have been identified using mass spectrometry. The results pointed out the presence of quercetin, myricetin and kaempferol derivatives (flavonols), a flavanone derivative, malic and quinic acids, hydroxycinnamic acids (*p*-coumaric and caffeic acids) and their derivatives as well as a galloyl derivative [48]. Additionally, two amino acids, L-tryptophan and methylentryptophan, have been detected in the chicory leaves.

Antioxidant and anti-inflammatory activities have been identified for aesculetin (**8**), a coumarin derivative identified in the chicory and *Sancho* tree. A recent study revealed that a naturally occurring aesculetin has been found to ameliorate the effects of urban coarse particulate matter inhalation by interfering with pulmonary inflammation and oxidative stress [49,50].

Some individual and total phenol concentrations of different parts of wild chicory have been determined using molecular absorption spectrometry and HPLC [51]. The flowers showed the highest total phenolic derivatives concentration (1531.2 mg Gallic acid equivalent (GAE)/100 g dry weight (d.w.)), followed by leaves (1180.3 mg GAE/100 g d.w.), roots (167.86 mg GAE/100 g d.w.) and stems (142.36 mg GAE/100 g d.w.). Additionally, the HPLC analysis indicated the presence of several phenolic acids, e.g., methyl gallic acid and ellagic acid (**12**), in the range of 30–50% from the total phenol concentration.

The phytochemical characterization of several edible purple-reddish vegetables has revealed that phenolic acids (chlorogenic and syringic acids) are the most abundant compounds [52]. The high content of phenolic derivatives, especially quercetin-3,4-*O*-diglucoside, has been found to be responsible for the higher antioxidant activities of red chicory.

**Figure 2:**
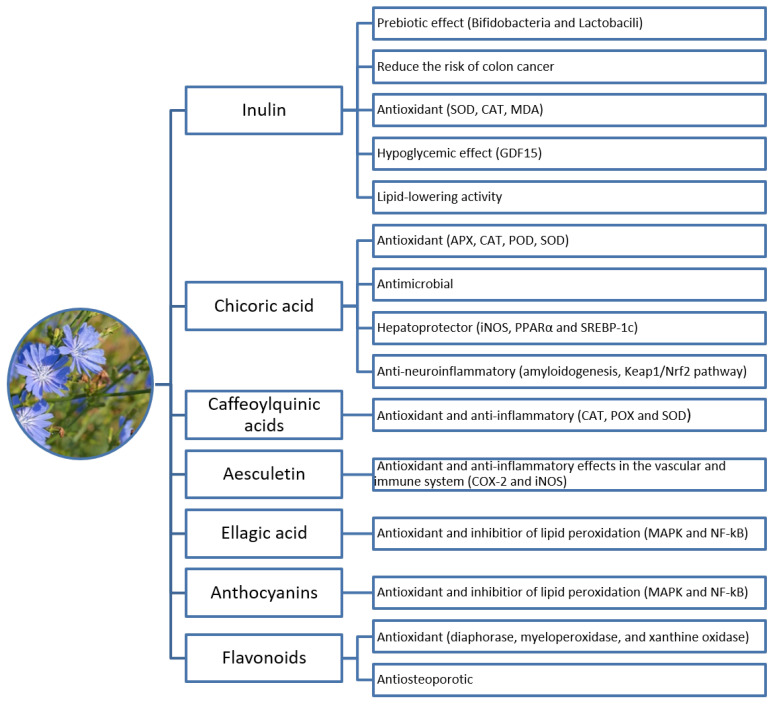
The physiological functions of *C. intybus* L. constituents.

The physiological functions and some of the action mechanisms of different *C. intybus* L. constituents are summarized in Figure 2.

### 2.1. Hairy Root Culture (HRC)

The ability of the roots of *C. intybus* L. species to produce a large variety of hydroxycinnamic acid derivatives is well known. Additionally, the hairy root culture (HRC) concept became a valuable technique for the biosynthesis of specialized metabolites. A comparison with the mother plant tissue revealed that HRC has a faster growth in a hormone-free environment. Apart from the common hydroxycinnamic acid derivatives, the HRC has the ability to produce metabolites not known in the mother plant roots [53,54]. Consequently, elicitation proved to be a useful method for the enhancement of metabolite production.

Among the large variety of compounds produced by the HRC of chicory, such as coumarins [55] and sesquiterpene lactones [56,57], caffeoylquinic acid derivatives are the major constituents [58], with dicaffeoylquinic acids of type **7** as major components. Among caffeoylquinic acids, di-CQAs are supposed to exhibit the most important antioxidant [59,60], antibacterial and anti-inflammatory [61] properties. For this reason, elicited hairy roots represents a feasible way for the biosynthesis of di-CQAs (about 12% of dry weight) [62]. Methyl jasmonate has been reported to improve the accumulation and production of CQAs in the HRC of *C. intybus* [63]. Elicitation has the unique capacity to induce the biosynthesis of 3,4,5-tricaffeoylquinic acid. It should be noted that tricaffeoylquinic acid derivatives have not previously been identified in the *C. intybus* roots.

Agrobacterium rhizogenes LBA 9402 transmuted the HRC of *C. intybus*, proved to be a good source of sesquiterpene lactones [64]. This modification also provided known hydroxycinnamates (caffeic, 5-CQA, 3,5-diCQA and 4,5-diCQA acids) and new glucosides, such as the sesquiterpene lactone 8-deoxylactucin glucoside and neolignan glucoside, or (7S,8R)-3′-demethyl-dehydrodiconiferyl alcohol-3′-*O*-*β*-glucopyranoside [41]. Agrobacterium strain K599 has been used for the genetic modification of two species of chicory [65]. As compared with the mother plant, the hairy roots showed an increased growth rate and revealed the accumulation of higher amounts of inulin.

The distribution of hydroxycinnamate and sesquiterpene lactone derivatives among the HRC of *C. intybus* L., the roots of wild and cultivated chicory, as well as the callus culture of the plant, have all been investigated [66]. The major antioxidant hydroxycinnamate in the hairy roots has been identified as 3,5-dicaffeoylquinic acid (**7**), with a 5.5% content reported for the dry weight. Regarding the presence of the sesquiterpene lactone derivatives in the hairy roots, it has been established that 85% of them are represented by the 8-deoxylactucin glucoside (crepidiaside A, **13**) and that 1.4% of them are based on the dry weight, almost two-fold more than in wild chicory roots.

### 2.2. Geographical Distribution

Different chicory ecotypes have been compared in terms of the phenolic profile and antioxidant properties [67]. Significant differences in phenolic acids (chicoric acid **5** and 5-caffeoylquinic acid **6**), flavonoid composition and antioxidant activity have been recorded among various ecotypes, as well as between the growing conditions (cultivated or wild plants). The total phenolic content has been found to be higher in cultivated ecotypes than in wild ones, whereas important amounts of flavonoids, especially apigenin-*O-*glucuronide, isorhamnetin-3-*O-*glucuronide and kaempferol-3-*O-*glucuronide, have been identified in commercial products.

An investigation on the bioaccessibility of chicory varieties in Turkey as well as the changes in anthocyanin, phenolics and antioxidant capacity have been reported [68]. The most frequent compounds in the methanol extracts have been identified as trans-ferulic and syringic acids (1.85 and 2.54 mg/kg, respectively), whilst the major flavanol was (+)-catechin. The highest flavanol content has been identified in the green chicory (0.62 mg/kg). Higher levels of extractable (8855.50 mg GAE/100 g) and hydrolysable (7005.51 mg GAE/100 g) phenolics and anthocyanin (12.80 mg/kg) have been determined in red chicory than in other varieties.

### 2.3. Agriculture Improvement

Arbuscular mycorrhizal fungi (AMF) are very common in earthly ecosystems, being well known due to their symbiotic associations with the roots of the vascular plants [69]. The AMF have been found to promote growth of the plant and to enhance its chemistry, morphology and structure [70]. The fungi-colonized roots also increase resistance to pathogens, influence the rhizosphere microbial composition and reduce environmental toxicity [71,72].

The involvement of arbuscular mycorrhizal fungi in the production of secondary metabolites as well as the activity of enzymatic antioxidants of the plant in the presence of toxic metal (Cd, Pb, Zn) have been investigated [73]. A comparison between chicory inoculated with *Rhizophagus irregularis* and non-inoculated plants has been performed in the presence of the above metals and in non-polluted environments. Whilst no differences have been found between the concentration of hydroxycinnamates in chicory cultivated in polluted or non-polluted environments, a higher concentration of caftaric acid **4**, cichoric acid **5** and 3,5-dicaffeoylquinic acid **7** have been identified in AMF-inoculated plants. Moreover, regardless of the cultivation conditions, increased enzymatic activities of CAT, POX and SOD antioxidant enzymes have been identified in mycorrhizal plants.

The concentration dynamics of health-promoting compounds with nutritional value in *C. intybus* L. by using the arbuscular mycorrhizal symbiont *Funneliformis mosseae* have been evaluated [74]. It has been found that Zn and Fe uptake notably increased as the result of mycorrhizal colonization of chicory. At the same time, it has been found that the Cu concentration at the level of roots decreased. Furthermore, the high levels of Zn and Fe in mycorrhizal plants has been correlated with the accumulation of inulin, fructose and carotenoids, which suggested a relationship with the production of health-promoting molecules [75,76].

Impact of *calcareous substrata* on AMF evolution and on lipid peroxidation in *C. intybus* roots has been studied [77]. Thus, the oxidative damage of chicory plants grown in CaCO_3_-enriched soil has been assessed via the lipid peroxidation increase monitored via malondialdehyde (MDA) production and the POD antioxidant enzyme activities. It has been found that non-mycorrhizal roots present higher levels of MDA as compared to mycorrhizal roots. This investigation revealed the potential of arbuscular mycorrhizal symbiosis to prevent lipid peroxidation by increasing chicory tolerance to elevated levels of CaCO_3_.

The growth parameters and enzymatic antioxidant parameters of wild chicory have been evaluated under salt stress [78]. Mycorrhizal *C. intybus* L. revealed good tolerance to moderate soil salinity. The increase in the soil salinity has been accompanied by the decrease in malondialdehyde levels in roots and leaves as well as by the increase in the proline content, especially in roots. Moreover, the activity of APX, CAT, POD and SOD antioxidant enzymes has been intensified under salt stress conditions.

The biological effects of different kinds of fertilizers on cultivated chicory have been investigated in the presence and the absence of pesticides [79]. The antioxidants, including total polyphenols and flavonoids along with the antioxidant activity of total phenolic compounds and flavonoids, have been identified to be higher in chicory grown in soil treated with chemical fertilizer and no pesticide. Furthermore, the level of carotenoids and the inhibitory effects on HepG2 cells have been positively influenced by an eco-developed fertilizer group under both pesticide conditions.

Humic acid and vermicompost have been found to increase the amount of bioactive components of chicory and therefore the antioxidant activity of the herb [80]. The use of a combination of humic acid and vermicompost resulted in a considerable accumulation of caffeic acid against other phenolic components (2773 mg/100 g d.w.). Moreover, when vermicompost has been used alone, the quantity of ellagic acid in shoots has increased to an average value of 262.51 mg/100 g d.w.

### 2.4. Extraction of the Main Constituents

Extraction of biologically active compounds present in red chicory has been performed using response surface methodology and evaluated in terms of the effects of temperature (62.4 °C) and time (25 min) [81]. A nontoxic solvent has been used for the extraction of bioactive compounds from an aqueous acetic acid solution at a pH of 2.5. Considerable levels of ellagic acid (**12**) and anthocyanins (cyanidin-3-*O*-glucoside and cyanidin-3-*O*-(6-*O*-malonyl) glucoside, compound **13** derivatives) have been discovered in red chicory. The components of the extract exhibited antioxidant properties and a slight in vitro lipid peroxidation inhibition.

The orthogonal matrix technique has been employed for the extraction of *C. intybus* L. roots [82]. The matrix consists of a 24 h impregnation time in 70% ethanol, followed by three sonication steps at 300 W. These conditions proved to be the optimal combination for the improvement of the chicory extract yield, total phenolic content and antioxidant activity. The bioactive substances have been primarily detected as caffeoylquinic acids (e.g., **6**, **7**).

### 2.5. Waste Valorization

A huge challenge of this century is waste management and decreasing food waste. The so-called food by-products represent a recent approach that enables for the valorization of food wastes. It consists in the recovery of valuable components by using processing technologies [83].

In the case of chicory, the food by-product concept means that the increase in the amounts of polyphenols in the extracts resulted from chicory grounds. Important amounts of antioxidant polyphenols have been recovered using Amberlite XAD 16, a resin that exhibited good adsorption properties [84]. Desorption has been performed using 70% aqueous ethanol. An alternative way is the use of Amberlite XAD-2, a nonionic polymeric resin [85]. In this case, two desorption procedures have been considered using methanol and water, respectively. Water extraction provided a higher phenolic content in the purified extracts, the highest mean values being 300 mg of phenolic/g of dried extract.

The antioxidant phenolic profile of a lyophilizate red chicory extract has been characterized and compared with BHT [86]. The antioxidant properties of red chicory have been evaluated against the lipid peroxidation of different oils and to oxidative stress via monitoring gene expression. A pleiotropic protective effect on stress responsive genes has been recorded.

A comparison between water-based conventional and microwave-assisted extraction techniques has been reported [87]. The yields and antioxidant activity of aqueous extracts have been correlated with several variables: water/sample ratio, extraction time and temperature (in conventional extraction). The conventional extraction procedure of chicory wastes has provided the highest amounts of phenolic antioxidants.

Forced chicory roots, by-products of chicon agricultural production, can be valorized for their caffeoylquinic acid (3-CQA, 5-CQA and 3,5-di-CQA) content. The optimization of extraction conditions of caffeoylquinic acids has been reported. The optimal experimental extraction conditions have been obtained using 70% ethanol at 50 °C or pure water at 90 °C [88]. Using an accelerated solvent extraction procedure, the highest antioxidant activity has been achieved at 115 °C using 40% aqueous ethanol [89].

### 2.6. Miscellaneous

The effects of microwave cooking on the chicory leaves have been reported. Eighteen phenolic compounds have been found in chicory leaf extracts [90]. *p*-Hydroxybenzoic acid, 3-*O*- and 5-*O*-caffeoylquinic acids, 4-feruloylquinic acid, kaempferol-3-sophoroside and quercetin-di-glucoside were the major phenolic derivatives. The amount of these phenolic compounds along with lipid peroxidation and the total flavonoid content has significantly increased as a result of microwave cooking.

## 3. Health Benefits of Chicory Constituents

The phenolic profile and antioxidant activity have been evaluated for different colored varieties of chicory vegetables (red-spotted, heavily red, and green-colored) [91]. Whereas the red color is provided by cyanidin derivatives (e.g., **14**), all of the investigated chicories contain important quantities of hydroxycinnamic and hydroxybenzoic acids (e.g., **2**, **4**–**6**). The particular composition of red chicories gives them great peroxyl radical scavenging activity in terms of effectiveness and capacity.

The chicory leaf extracts demonstrated antioxidant activity in human plasma and an inhibitory effect on lipid peroxidation. A significant effect has also been identified on the level of thiol groups, a biomarker of oxidative stress [92]. These studies found that anthocyanins (compound **14** derivatives) are the main phenolic compounds in red chicory leaves, which are responsible for their antioxidant activity.

The antioxidant properties of polyphenols contained by minimally processed red chicory products (storage at 4 °C) have been investigated in model reactions catalyzed using enzymatic sources of reactive oxygen species: diaphorase, myeloperoxidase and xanthine oxidase [93]. It has been found that less than 20% of flavonoids and hydroxycinnamic acids have been lost during storage of the processed red chicory extracts. The model enzymatic reactions indicated that generation of the superoxide radical along with quinonic, hypochlorite and hydrogen peroxide radicals are responsible for the strong antioxidant activity of the extracts.

The enzymatic activity of peroxidase and polyphenol oxidase in *C. intybus* L. leaves has been found to be temperature-dependent. Moreover, the antioxidant behavior of the phenolic components of chicory leaves has been affected by the applied drying techniques [94]. The freeze-dried and hot air-dried leaves have preserved the most of the phenolics and thus these possess better antioxidant activity.

The anti- and pro-oxidant activity of water extracts of chicory have been analyzed [95]. The linoleic acid-*β*-carotene micellar model system has been used to investigate the antioxidant activity of juices obtained at 2 °C and of those heated at 102 °C. Both anti- and pro-oxidant activity have been identified for the juice obtained at 2 °C. The consequences of thermal treatments such as freeze-drying, freezing and boiling have been reported [96]. It has been found that in a cold medium, all juices contain a thermally unstable component, which promoted and accelerated linoleic acid peroxidation [97]. The presence and the activity of thermally stable antiperoxyl radical components, which confer protection from peroxidation to linoleic acid, are masked by the presence of these pro-oxidant compounds. The latter can be removed via separation or inactivated via heating.

The antioxidant properties of *C. intybus* L. extract embedded in chitosan nanocomposite nanofibers and silver nanoparticles using chicory leaf-derived callus extract have been reported [98,99]. In addition, a combination of chitosan, polyvinyl alcohol and 50 mg of chicory root extract exhibited the most efficient antioxidant and antibacterial activity. The highest yield in the total phenolic content of root extracts (4 mg GAE/g) has been obtained using ethanol as a solvent.

The murine model and nutrigenomic and metagenomic analyses have been used for testing the water extracts of chicory roots. Novel mechanisms of action as antioxidant, antiviral and antibacterial, as well as on cancer prevention have been disclosed [100].

The antiosteoporotic effect of *C. intybus* in glucocorticoid-induced osteoporosis in rats has been investigated [101]. The antioxidant properties of chicory water extracts (flavonoids and inulin content) have increased the mineral bone density in the dexamethasone-treated group.

The effects of a chicory-supplemented diet against oxidative stress and hepatic disorders induced by chlorpromazine and sodium nitrite (nitrosamine precursors) have been studied in male rats [102]. It has been found that this approach can decrease the hepatotoxicity and oxidative stress induced by nitrosamine precursors due to its efficient scavenging free radicals responsible for cell damage.

Novel heteropolysaccharides have been isolated from *C. intybus* L. roots [103]. Their preliminary structural characterization has indicated a combination of glucitol, fructose and glucose along with sorbin in a ratio of 10:14:6:1 and with a molecular weight of 8511.4 Da. These chicory polysaccharides showed reduced lipid activity in male rats with non-alcoholic fatty liver disease.

The acute alcohol-induced steatosis in mice can be decreased using chicoric acid through a mechanism involving the inducible nitric oxide synthase (iNOS) and iNOS-dependent signaling cascades in the liver [104].

The impact of chicory seed extract on hepatic steatosis determined by different stages of diabetes in rats and established in HepG2 cells via the bovine serum albumin–oleic acid complex has been evaluated [105]. Simultaneous treatment with *C. intybus* L. has prevented steatosis, inflammation and fibrosis of the cells and tissues. Treatment with *C. intybus* L. alone has reestablished the normal levels of the hepatic proteins and increased the expression of *PPARα* and *SREBP-1c* genes. Thus, chicory extract exhibits *PPARα* agonist properties. Additionally, chicory seed extract has been found to release glycerol from HepG2 cells.

It has been reported that the oxidative stress induced by methotrexate in rats can be reduced by using a hydroalcoholic root extract of *C. intybus* L. [106]. The hepatoprotective effect of chicory syrup against liver damage, lipid peroxidation and oxidative stress induced by a pyrethroid insecticide, deltamethrin, has been investigated in male rats [107]. The results of chicory syrup administration consist in decreasing of lipid peroxidation level, increasing the activity of antioxidant enzymes and improving the histopathological profile of the liver. *C. intybus* L. has also been found to provide hepatic protection against cirrhosis induced by thioacetamide through intracellular pathways: corrections in the liver functions, inflammatory and redox conditions [108].

Along with the antioxidant and antimicrobial properties, extracts from chicory roots exhibited toxigenic properties against fungi. The experiments on rats indicated that the use of the chicory root extract may enhance the generation of functional products that could be able to improve aflatoxins-induced oxidative stress in liver [109].

The impact on the large intestinal mucosa induced by the introduction of small amounts of chicory inulin in growing pigs’ diet has been investigated [110]. Native inulin-enriched diets have been found to improve the levels of antioxidant proteins, protein foldases and the profile of molecular chaperones. These proteins play an important role in preserving the integrity of mucosal cells and confer protection against reactive oxygen species and some endotoxins.

The impact on the intestinal health of nutraceutical phytochemicals presented in Treviso red chicory has been described [111,112]. The presence of antioxidant anthocyanins in this chicory variety has a direct scavenging impact on the formation of reactive oxygen species over the gastrointestinal tract. The impact on intestinal health appears to be enhanced by the good ability of anthocyanin metabolites to be diffused in intestinal tissue. It has been suggested that the protection of the lining of the gut for an efficient intestinal barrier may arise from the interaction between anthocyanin derivatives and the mucopolysaccharide complexes.

The antioxidative profile of the body along with the antibacterial activity of *β*-glucuronidase at the level of the gut have been enhanced by a combination of monocaffeoylquinic and dicaffeoylquinic acids [113]. This mixture has been extracted from the seeds, roots and root peels of *C. intybus* L. Rats’ diets improved with chicory root extracts containing different amounts of fructan and polyphenols triggered positively physiological effects to them, especially in terms of the blood lipid profile [114,115]. Positive modifications in the lipid profile of the blood serum have been induced by a chicory leaf extract. The extract has been found to be rich in chicoric and chlorogenic acids and polyphenolic glycosides.

Natural chicoric acid extracts might have a significant impact on diabetes treatment since their administration to diabetic rats has been reported to reduce basal hyperglycemia and improve tolerance to glucose [116,117]. Moreover, the effects of chicoric acid on regulating insulin resistance and chronic inflammatory responses have been explored by glucosamine in HepG2 cells [118]. It resulted in a decrease in the reactive oxygen species levels (COX-2 and iNOS).

Diabetic obese albino mice have been treated with soluble root extracts of chicory and the effects on the body weight and blood glucose level as well as the anti-inflammatory and antioxidant status have been monitored [119]. The positive results of chicory root administration appear to be due to the upregulating of the GDF-15 level.

The enhancement of the mitochondrial function and of energy metabolism as a result of chicoric acid administration has been reported to ensure neuron survival against inflammation [120]. Recent results have indicated that a chicoric acid-based nutritional supplementation has ameliorated cognitive impairment induced by D-galactose, as well as the SH-SY5Y cell apoptosis induced by hydrogen peroxide via promotion of the Keap1/Nrf2 signal pathway and its antioxidant enzymes. These findings pointed out the high potential of the nutritional preventive strategy based on chicoric acid for the use against oxidative stress-related cognitive impairment [121,122].

The inhibitory effects of chicoric acid on systemic inflammation-induced neuroinflammation, amyloidogenesis and cognitive impairment have been investigated [123]. It has been found that chicoric acid prevented lipopolysaccharide-induced memory impairment and neuronal loss through behavioral tests and histological examination. Moreover, chicoric acid downregulated lipopolysaccharide-induced glial overactivation by inhibiting the MAPK and NF-kB pathway.

## 4. Conclusions

This comprehensive review highlights the health benefits of *Cichorium intybus* L.’s main constituents. It covers their occurrence, natural biosynthesis, geographical distribution and waste valorization. The antioxidant properties of chicory components, first reported in 1995 [124], are also summarized. The present literature survey also suggests that the chicory herb parts exhibit numerous biological activities such as anti-inflammatory, antimicrobial, hepatoprotective, antidiabetic, gastroprotective, analgesic, tumor-inhibitory and antiallergic effects. Further in vitro and in vivo studies are required in order to evaluate the possible therapeutic application of this herb.

## Data Availability

All data used in this study were gathered from open literature sources or scientific journals available under institutional subscription. Data sharing is not applicable to this article.

## Abbreviations

APX	Ascorbate peroxidase
BHT	Butylated hydroxytoluene
Caco-2	Human colon carcinoma cell line
CAT	Chloramphenicol acetyltransferase
COX	Cyclooxygenase
3-CQA	3-Caffeoylquinic acid (chlorogenic acid)
4-CQA	4-Caffeoylquinic acid
5-CQA	5-Caffeoylquinic acid
3,4-diCQA	3,4-Dicaffeoylquinic acid
3,5-diCQA	3,5-Dicaffeoylquinic acid (isochlorogenic acid)
4,5-diCQA	4,5-Dicaffeoylquinic acid
DPPH	2,2-Diphenyl-1-picrylhydrazyl
GAE	Gallic acid equivalents
GDF15	Growth differentiation factor 15
HepG2	Liver hepatocellular carcinoma
HRC	Hairy root culture
MAPK	Mitogen-activated protein kinase
MDA	Malondialdehyde
iNOS	Inducible nitric oxide synthase
NF-κB	Nuclear factor kappa B
POD	Antioxidant enzyme peroxidase
POX	Class III plant peroxidase
PPARα	Peroxisome proliferator-activated receptor alpha
mRNA	Messenger RNA
SOD	Superoxide dismutase
SREBP-1	Sterol regulatory element-binding protein 1
